# The Functional Characterization of Long Noncoding RNA *SPRY4-IT1* in Human Melanoma Cells

**DOI:** 10.18632/oncotarget.1863

**Published:** 2014-03-26

**Authors:** Joseph Mazar, Wei Zhao, Ahmad M. Khalil, Bongyong Lee, John Shelley, Subramaniam S. Govindarajan, Fumiko Yamamoto, Maya Ratnam, Muhammad Nauman Aftab, Sheila Collins, Brian N. Finck, Xianlin Han, John S. Mattick, Marcel E. Dinger, Ranjan J. Perera

**Affiliations:** ^1^ Sanford-Burnham Medical Research Institute, Orlando, FL 32827, USA; ^2^ Department of Genetics and Genome Sciences, Case Western Reserve University School of Medicine, Cleveland, OH 44106, USA; ^3^ Division of Geriatrics and Nutritional Sciences, Washington University School of Medicine, St. Louis, MO 63110, USA; ^4^ Garvan Institute of Medical Research and St Vincent's Clinical School, University of New South Wales, Darlinghurst NSW 2010, Australia

**Keywords:** long noncoding RNA, melanoma

## Abstract

Expression of the long noncoding RNA (lncRNA) SPRY4-IT1 is low in normal human melanocytes but high in melanoma cells. siRNA knockdown of SPRY4-IT1 blocks melanoma cell invasion and proliferation, and increases apoptosis. To investigate its function further, we affinity purified SPRY4-IT1 from melanoma cells and used mass spectrometry to identify the protein lipin 2, an enzyme that converts phosphatidate to diacylglycerol (DAG), as a major binding partner. SPRY4-IT1 knockdown increases the accumulation of lipin2 protein and upregulate the expression of diacylglycerol O-acyltransferase 2 (DGAT2) an enzyme involved in the conversion of DAG to triacylglycerol (TAG). When SPRY4-IT1 knockdown and control melanoma cells were subjected to shotgun lipidomics, an MS-based assay that permits the quantification of changes in the cellular lipid profile, we found that SPRY4-IT1 knockdown induced significant changes in a number of lipid species, including increased acyl carnitine, fatty acyl chains, and triacylglycerol (TAG). Together, these results suggest the possibility that SPRY4-IT1 knockdown may induce apoptosis via lipin 2-mediated alterations in lipid metabolism leading to cellular lipotoxicity.

## INTRODUCTION

Eukaryotic genomes express a complex repertoire of thousands of RNAs that lack protein-coding capacity [1, 2]. These noncoding RNAs (ncRNAs) are broadly classified as long or small based on a nucleotide length of >200 or <200 nucleotides (nt), respectively. Small regulatory ncRNAs are commonly conserved and are involved in transcriptional and posttranscriptional gene regulation through specific base pairing with their target genes or transcripts. In contrast, long noncoding RNAs (lncRNAs) are less well conserved and regulate gene expression by diverse mechanisms, including epigenetic mechanisms, that remain incompletely understood [3-6]. LncRNAs may be derived from genomic sequences that are intergenic, intronic, overlapping, or antisense with respect to nearby protein-coding genes, and although some lncRNAs may be translated into short polypeptides, the vast majority are rarely or never translated [7, 8].

Until recently, lncRNAs were frequently dismissed as non-functional transcriptional “noise” [9]. However, the past few years have seen a rapid increase in our understanding of the regulatory functions of lncRNAs and their role in human disease and development [10, 11]. LncRNAs exhibit exquisite context-dependency commensurate with their presumed regulatory role, with cell type-specific expression and localization to discrete subcellular compartments [12-14]. At the molecular level, lncRNAs influence target gene expression at specific genomic loci by directly interacting with chromatin regulatory proteins and/or by modulating the activity of their interacting partners [15-20]. LncRNAs can function as decoys for bound proteins and can also alter the structure and function of the protein [17]. Although lncRNAs play physiological roles during normal cellular development and differentiation [21], changes in their expression are associated with several diseases, including cancer, heart disease, Alzheimer's disease, psoriasis, and spinocerebellar ataxia type 8 [22]. Accumulating evidence suggests that lncRNAs may also play a role in tumorigenesis. For example, increased expression of *HOTAIR* is associated with poor prognosis in pancreatic cancer [23], and increased expression of *PCGEM1* and *PCA3/DD3* is associated with a high risk of developing prostate cancer [24].

Recently, we identified a number of lncRNAs that are differentially expressed in melanoma cell lines relative to melanocytes and keratinocytes [14, 25]. One of these, *SPRY4-IT1* (*Sprouty4-Intron 1*; GenBank accession ID AK024556), is highly expressed in melanoma cells relative to melanocytes, and is localized predominantly in the cytoplasm. Previously we showed that *SPRY4-IT1* is derived from the intronic region of the *SPRY4* gene and that its predicted secondary structure contains several long hairpins [14]. Moreover, RNAi-mediated knockdown of *SPRY4-IT1* inhibited invasion and proliferation and induced apoptosis of melanoma cells, suggesting an important role for this lncRNA in melanoma biology.

In the present study, we sought to identify *SPRY4-IT1*-interacting proteins and elucidate this lncRNA's molecular function. In melanoma cells, *SPRY4-IT1* transcripts are processed in the nucleus prior to transport to the cytoplasm, where they are primarily located in polysomes. We also identify the phosphatidate lipin 2 as a major *SPRY4-IT1*-binding protein, and demonstrate the existence of a novel lipid regulatory mechanism involving *SPRY4-IT1* and lipin 2. Our results are consistent with the possibility that *SPRY4-IT1* knockdown may induce apoptosis via lipin 2-mediated alterations in lipid metabolism leading to cellular lipotoxicity. Together these results provide novel insight into the mechanisms by which extranuclear processing of lncRNAs contributes to melanoma biology.

## RESULTS

### Processing of SPRY4-IT1 Transcripts

**Figure 1:**
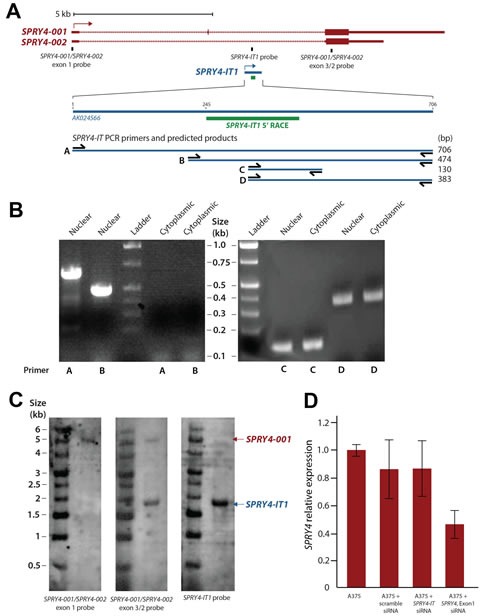
Maturation and Cellular Compartmentalization of SPRY4-IT1 Transcripts (A) The sequence and location of *SPRY4* and *SPRY4-IT1*. *SPRY4-IT1* is embedded in the *SPRY4* parent transcript. *SPRY4-IT1* starts in the first intron of the *SPRY4* gene and extends up to exon 3. 5′ RACE shows the maturation and cleavage of a 244 nt transcript in the 5′ region of *SPRY4-IT1*. (B) Detection of nuclear and cytoplasmic forms of *SPRY4-IT1.* (C) Northern blot analysis shows the sizes of *SPRY4* (4.9 kb) and *SPRY4-IT1* (1.8 kb). The *SPRY4* exon 1 probe hybridizes specifically to *SPRY4*, but the *SPRY4* exon 3 probes recognize both *SPRY4* and *SPRY4-IT*. (D) *SPRY4* exon 1 siRNA knocks down the expression of *SPRY4,* but not *SPRY4-IT1,* in A375 melanoma cells.

*SPRY4-IT1* was originally identified as a 706 bp transcript in adipose tissue as part of a large-scale cDNA sequencing study [26]. The *SPRY4-IT1* sequence was shown by PCR to initiate in intron 1 of the *SPRY4* gene and extend to exon 3 (Fig. 1A). Interestingly, the 5′ region of the full-length *SPRY4-IT1* transcript was detected in samples of nuclear, but not cytoplasmic RNA, suggesting that the processing of *SPRY4-IT1* takes place before nucleocytoplasmic export (Fig. 1B). We confirmed using 5′ RACE that the first 244 nt of the 5′ sequence is missing from cytoplasmic *SPRY4-IT1* (Fig. S1). To validate this observation and to visualize the subcellular localization of *SPRY4-IT1*, we designed several RNA-FISH probes specific for the 3′ or 5′ regions of *SPRY4-IT1*. This analysis confirmed the presence of the full-length transcript in the nucleus but not in the cytoplasm (Fig. S2), in agreement with PCR results. *SPRY4-IT1* staining in melanocytes is mainly localized to the nucleus and this may be the reason of intense nuclear staining in melanocytes. Together these data demonstrate that the *SPRY4-IT1* transcript undergoes maturation by cleavage of the 5′ region prior to its transport to the cytoplasm. This may suggest that the regulation of the 5′ cleavage controls the export of the transcript to the cytoplasm thus, the overall SPRY4-IT1 content may depend on the efficiency of the nuclear excision event.

Although the precise 3′ termination site of the *SPRY4-IT1* transcript is not yet known, northern blot analysis shows a *SPRY4-IT1* transcript size of approximately 1.8 kb (Fig. 1C), and the 3′ sequence of the transcript is very similar to that of Exon 3 of the *SPRY4* gene. Therefore, we propose that *SPRY4-IT1* carries both intronic and exonic sequences and can be considered a noncoding splice variant of the *SPRY4* gene.

### SPRY4-IT1 and SPRY4 Gene Transcripts Function Independently

**Figure 2:**
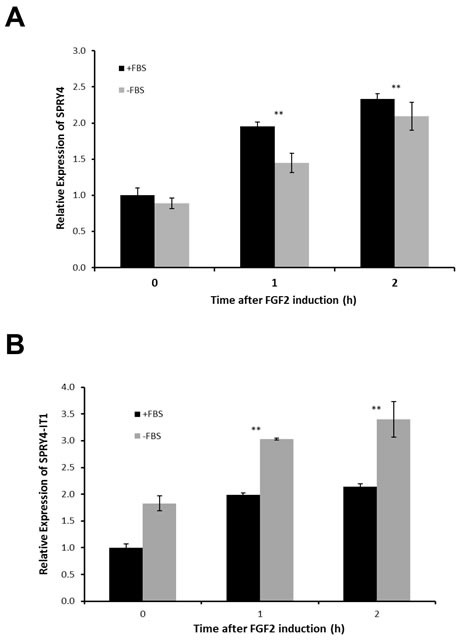
SPRY4 and SPRY4-IT1 Are Coordinately Regulated in Melanocytes Relative expression of *SPRY4-IT1* and *SPRY4* following induction by FGF2. Melanocytes were treated with 10 ng/ml FGF2 for 1 or 2 h in the presence or absence of FBS, and the fold change in expression of *SPRY4* (A) and *SPRY4-IT1* (B) was measured by qPCR

We next asked whether the transcription and function of *SPRY4-IT1* and *SPRY4* are coordinately or independently regulated. Despite the similar expression patterns of *SPRY4* and *SPRY4-IT1*, siRNA-mediated knockdown of *SPRY4* (siRNA targeting exon 1) in A375 melanoma cells reduced the expression of *SPRY4* but not *SPRY4-IT1* (Fig. 1D), demonstrating that transcription of the coding (*SPRY4)* and noncoding (*SPRY4-IT1*) genes occurred independently. To confirm this in a more physiological setting, we treated melanocytes (low expression of SPRY4-IT1) with fibroblast growth factor 2 (FGF2), which is known to induce *SPRY4* expression [27]. In serum-containing medium, FGF2 induced expression of *SPRY4-IT1* and *SPRY4* to similar extents (Fig. 2A). However, *SPRY4-IT1* transcription was further elevated in melanocytes treated with FGF2 in serum-free medium (Fig. 2B), confirming independent transcription of *SPRY4-IT1* and *SPRY4*. These results suggest that SPRY4-IT1 responds to starvation and general stress responses.

To determine whether the stability of *SPRY4-IT1* and *SPRY4* transcripts is independently regulated, we treated A375 cells with the polymerase II transcriptional inhibitor α-amanitin and measured the transcript levels by RT-PCR. We observed that *SPRY4* RNA decayed faster than *SPRY4-IT1* in both the nucleus and cytoplasm, supporting the functional independence of the transcripts (Fig. S3). Finally, we independently knocked down *SPRY4* and *SPRY4-IT1* in A375 cells using transcript-specific siRNAs. Confirming our previous report [14], A375 cell invasion was inhibited ~50% by *SPRY4-IT1* knockdown but was unaffected by *SPRY4* silencing (Fig. S4A). Similarly, *SPRY4-IT1* silencing induced apoptosis of A375, as measured by caspase 3 activity, more effectively than did *SPRY4* knockdown (Fig. S4B). Collectively, these data establish the transcriptional and functional independence of *SPRY4-IT1* and its host gene *SPRY4*.

**Figure 3:**
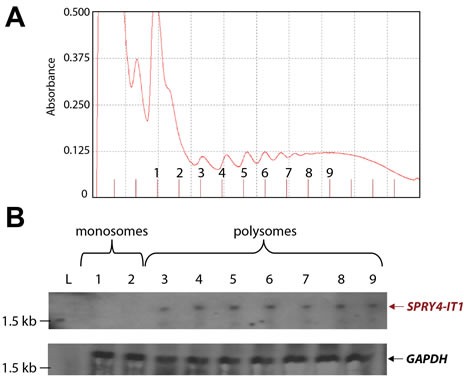
SPRY4-IT1 Accumulates in Polysomes and Is Absent from Monosomes (A) Density gradient fractionation of monosome and polysome peaks. (B) Northern blot analysis showing *SPRY4-IT1* probe hybridization to RNA isolated from polysomes but not monosomes. A GAPDH probe served as a control. Separate blots were prepared to probe SPRY4-IT1 and control (GAPDH) due to concerns of overlap in fragment size. L= RNA size marker.

### SPRY4-IT1 Is Localized to the Polysome Fraction and Binds the Lipid Phosphatase Lipin 2

In a recent ribosomal footprint study, Ingolia et al. [28] reported that cytoplasmic lncRNAs are primarily found in ribosomal clusters or polysomes. To determine the location of *SPRY4-IT1*, we used density gradient centrifugation to isolate monosomal and polysomal fractions from A375 cells and recovered 13 fractions: 2 monosomal and 11 polysomal (Fig. 3A). Total RNA was isolated from the individual fractions and analyzed for the presence of *SPRY4-IT1* by northern blotting. *SPRY4-IT1* was detected only in polysomal fractions 3 through 9 (Fig. 3B), indicating that *SPRY4-IT1* is indeed associated with polysomes and is not retained in monosomes, consistent with the findings of Ingolia et al. [28]. Sequence analysis further confirmed that *SPRY4-IT1* does not show any coding potential; understanding its role in possible translational regulation is an ongoing study in our laboratory.

**Figure 4:**
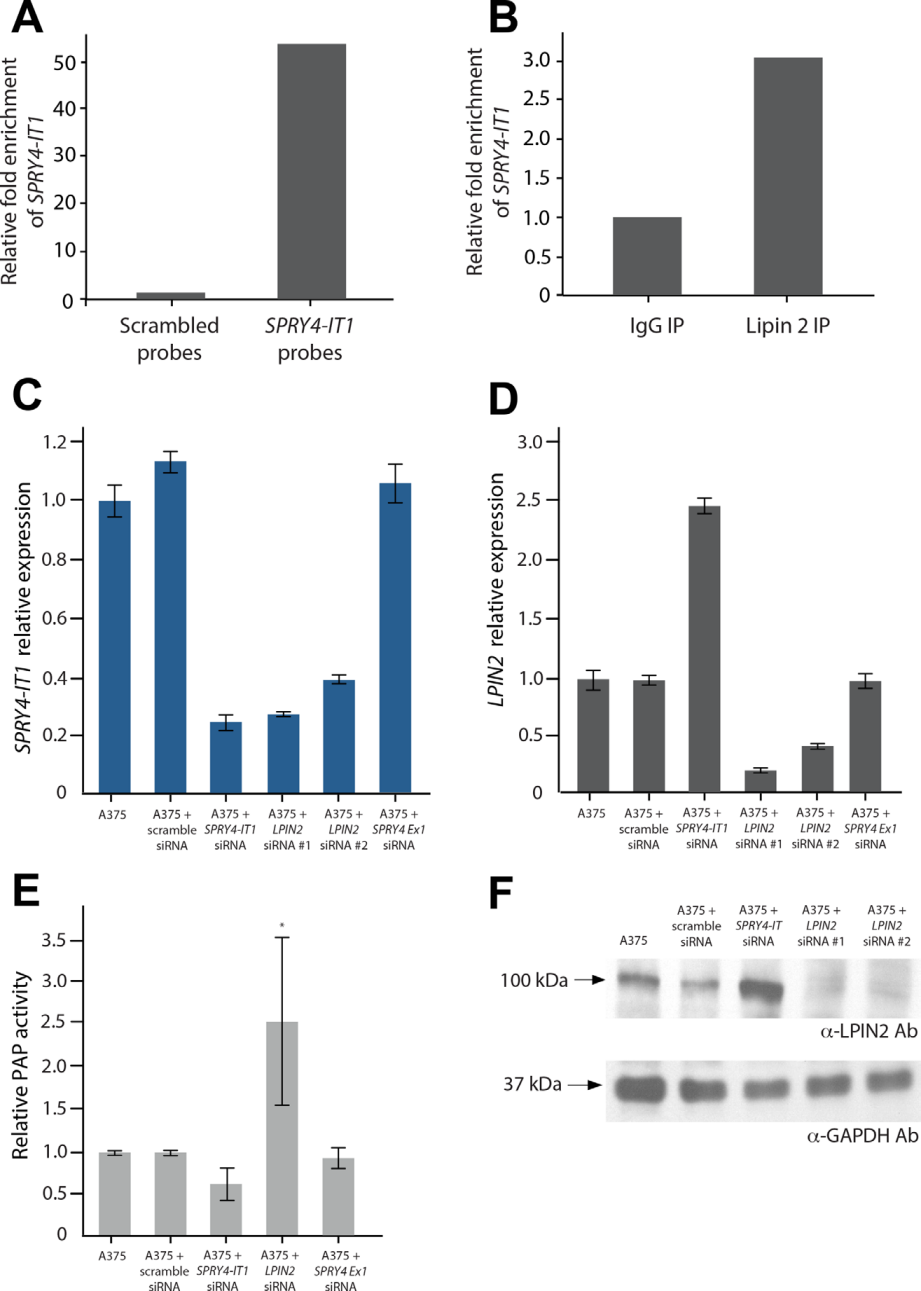
SPRY4-IT1 Knockdown Increases Lipin 2 Protein Accumulation in Melanoma Cells A) Affinity purification of *SPRY4-IT1* from A375 cell lysates with *SPRY4-IT1*-specific probes followed by qPCR. *SPRY4-IT1* is enriched compared to scrambled (control) probes. U1 RNA was used as endogenous control for pull-downs. (B) qPCR validation showing enrichment of *SPRY4-IT1* following immunoprecipitation of A375 cell lysates with lipin 2-specific or control IgG antibodies. (C) Relative expression of *SPRY4-IT1* in lipin 2 knock-down cells (D) Relative expression of lipin 2 after siRNA-mediated knockdown of *SPRY4-IT1*, *SPRY4* exon 1 or lipin 2. (E) Phosphatidic acid phosphatase assay. (F) Western blot analysis of lipin 2 following siRNA-mediated knockdown of *SPRY4-IT1* or lipin 2. All experiments were performed in triplicate.

We next sought to identify *SPRY4-IT1*-binding proteins in melanoma cells. For this, we affinity purified endogenous *SPRY4-IT1* from crosslinked A375 lysate (Fig. 4A) and interrogated the *SPRY4-IT1*-associated proteins by mass spectrometry (MS). A group of candidate proteins was identified and quantified by spectral counting (Table S1). The most abundant protein candidate was lipin 2 [29], a phosphatidic acid phosphatase (PAP) that converts phosphatidate into diacylglycerol (DAG). Lipin 2 was pulled down specifically with *SPRY4-IT1* but not with scrambled (control) probes. To verify the physical association between lipin 2 and *SPRY4-IT1*, lipin 2 was immunoprecipitated, and associated RNAs were isolated and interrogated for the presence of *SPRY4-IT1* by qPCR. As shown in Figure 4B, *SPRY4-IT1* transcripts were enriched in anti-lipin 2 immunoprecipitated compared with control IgG.

We next investigated the functional significance of the association between *SPRY4-IT1* and lipin 2 in melanoma cells using gene-specific RNAi. Knockdown of lipin 2 with two independent siRNAs led to a concurrent loss of *SPRY4-IT1* (Fig. 4C). This result suggests that the loss of lipin 2 most likely destabilizes *SPRY4-IT1*. In contrast, knockdown of *SPRY4-IT1* increased both lipin 2 mRNA and protein by ~2.5-fold (Fig. 4D and 4F). PAP enzymatic activity was markedly increased in *SPRY4-IT1* knockdown cells (Fig. 4E). Given that *SPRY4-IT1* knockdown had no effect on lipin 1 expression (Fig. S5), these data suggest that the increased PAP activity in these cells is due to the increased abundance of lipin 2. It is worth noting that knockdown of the *SPRY4* gene did not affect the expression of either *SPRY4-IT1* or lipin 2 (Fig. 4).

### SPRY4-IT1 Knockdown Increases Triacylglycerol Production via Lipin 2

**Figure 5:**
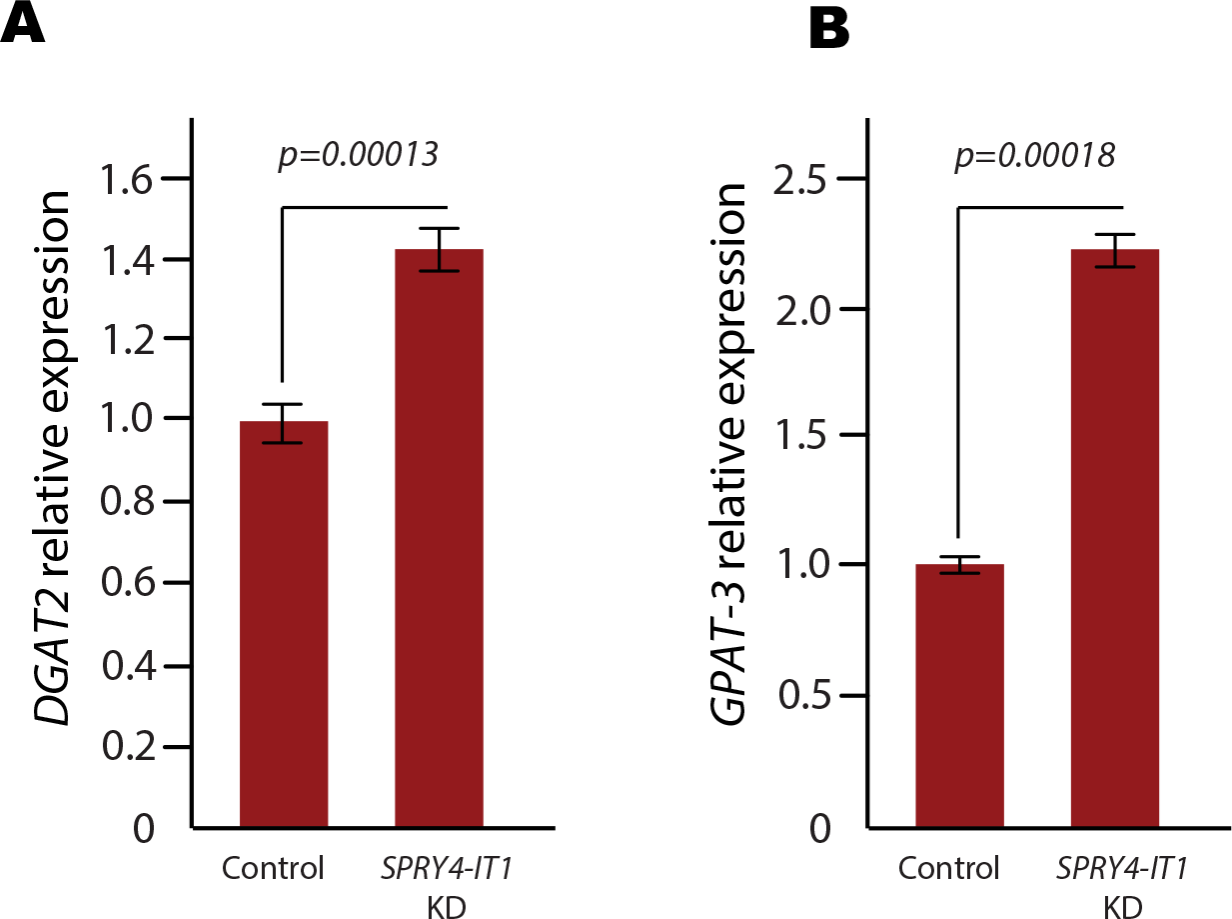
Expression of Diacylglycerol O-acyltransferase 2 and (DGAT2) Acyl-coA:glycerol-3-phosphate Acyltransferase 3 (GPAT3) Following SPRY4-IT1 Knockdown Both DGAT2 (A) and GPAT3 (B) mRNA levels are increased upon knockdown of SPRY4-IT1 in A375 cells, which may explain the decrease in DAG levels and increase in TAG levels observed in these cells (Table S2).

To investigate the effect of *SPRY4-IT1* modulation of lipin 2 expression on global lipid metabolism in melanoma cells, we subjected *SPRY4-IT1* knockdown and control A375 cells to shotgun lipidomics, an MS-based assay that permits the quantification of changes in the cellular lipid profile (Table S2). We found that *SPRY4-IT1* knockdown induced significant changes in a number of lipid species, including increased acyl carnitines (+80.21%), fatty acyl chains (+14.24%), and triacylglycerol (TAG) (+15.02%), as well as decreased phosphatidic acid (-37.72%), phosphatidylcholine (-35.58%), phosphatidylinositol (-27.78%), and phosphatidylserine (-26.40%). Interestingly, DAG content was decreased despite the increase in TAG levels. Since DAG is required for TAG synthesis, we hypothesized that the reduction in DAG levels in *SPRY4-IT1* knockdown cells may be due to efficient conversion of DAG to TAG. To test this, we examined the expression of acyl-CoA:glycerol-3-phosphate acyltransferase 3 (GPAT3), diacylglycerol O-acyltransferase 1 (DGAT1), and diacylglycerol O-acyltransferase 2 (DGAT2), since these enzymes play essential roles in DAG to TAG conversion. qPCR analysis showed that both DGAT2 and GPAT3 mRNA levels were increased in *SPRY4-IT1* knockdown cells (Fig. 5), providing a possible mechanism for the changes in DAG and TAG levels in these cells. We postulate that one mechanism by which *SPRY4-IT1* knockdown induces apoptosis could be through lipin 2-mediated lipotoxicity. To investigate further, we knocked-down and over-expressed lipin 2 in A375 cells and measured cell invasion efficiency and doubling time. Interestingly, we observed that cell doubling time was increased and cell invasion was decreased in both knock-down and force-expressed cells (Fig. S6A and B). We postulate that lipin 2 levels must be maintained on a steady state level in melanoma cells, and modulating lipin 2 levels (either an increase or decrease) can negatively affect cell physiology. For comparison, we subjected the primary melanocyte cell line HEM-l to shotgun lipidomics, and compared cellular lipid profiles to A375 cells. Interestingly, we found that lipid levels were significantly different in these two cell types. For an example, phosphatidylethanolamine (PE), Phosphatidylserine (PS), Phosphatidic acid (PA), Sphingomyelin (SM) were higher in melanocytes, compared to melanoma cells (Table S3). Studying the role of lipids in melanocyte and melanoma biology is an ongoing study in our laboratory.

## DISCUSSION

In contrast to small ncRNAs, which are highly conserved and play a role in transcriptional and posttranscriptional gene silencing through specific base pairing, lncRNAs are often poorly conserved (perhaps reflecting lineage specificity and/or more plastic structure–function constraints) and regulate gene expression by diverse mechanisms that are not yet fully understood. We previously identified the lncRNA *SPRY4-IT1,* which is derived from the intronic region of the Sprouty4 gene, and is upregulated in human melanomas compared to normal melanocytes and keratinocytes [14]. siRNA-mediated knockdown of *SPRY4-IT1* in melanoma was shown to affect cell proliferation and apoptosis, suggesting that high *SPRY4-IT1* expression may play an important role in the molecular etiology of human melanomas. In this study, we sought to probe this further by identifying the molecular function of *SPRY4-IT1* and elucidating its possible molecular function in melanomas.

We found that *SPRY4-IT1* is part of a longer noncoding transcript that is processed by cleavage of a 244 nt 5′ fragment prior to transport to the cytoplasm. The fate of the 244 nt fragment is unknown, but it is likely to be degraded by exo- and endonucleases. The maturation of *SPRY4-IT1* is similar to the nuclear processing of *MALAT1* in lung cancer [30]. We also showed that *SPRY4* and *SPRY4-IT1* are independent transcripts, although they may be regulated by the same transcriptional machinery. A recent study classifying lncRNAs according to their genomic origin showed they may originate from (a) a bidirectional promoter adjacent to a protein-coding gene, (b) the 5′ UTR, (c) a retained intron, (d) within an intron, (e) the 3′ UTR, (f) an intergenic non-protein-coding region, (g) antisense to protein-coding genes, or (f) a combination of the above [31]. According to this classification, *SPRY4-IT1* therefore belongs to the group of intron-retained lncRNAs.

One of the direct targets of *SPRY4-IT1* identified in this study is lipin 2, a member of the lipin family of phosphatidate phosphatase enzymes [32]. The mouse lipin 2 protein has previously been shown to be post-transcriptionally regulated [33]. We showed that knockdown of *SPRY4-IT1* modulates cellular concentrations of lipin 2 substrates, including phosphatidate, and cells expressing high *SPRY4-IT1* levels, such as melanomas, might thus be expected to accumulate lipin 2 substrates. Indeed, increased levels of phosphatidate have been linked to certain types of cancers through allosteric activation of the molecular target of rapamycin (mTOR) [34] and to a possible role for lipin 2 in statin resistance of colorectal carcinoma cells *in vitro* [35]. It would be of interest to determine whether the influence of *SPRY4-IT1* on melanocyte and melanoma proliferation involves inhibition of lipin 2 activity and/or activation of mTOR.

Our results are the first to demonstrate a role for a lncRNA in lipid metabolism in melanoma cells, and collectively, the data support the notion that *SPRY4-IT1* may play a critical role in melanocytic transformation.

## MATERIAL AND METHODS

### Cell Lines

Human epidermal melanocytes (HEM-l) were grown in MelM media containing MelGS growth supplements, 0.5% fetal bovine serum (FBS), penicillin, and streptomycin. Melanoma cells A375 (stage 4) were grown in Complete Tu medium containing a 4:1 mixture of MCDB-153 medium with 1.5 mg/ml sodium bicarbonate and Leibovitz's L-15 medium with 2 mM L-glutamine, 2% FBS, and 1.68 mM CaCl2. Cells were grown at 37°C in a humidified 5% CO2 atmosphere. All cell lines were obtained from ATCC (http://www.atcc.org).

### Cloning and Identification of 3′ Spliced Forms of SPRY4-IT1

Total RNA was isolated from A375 cells with TRIzol (Invitrogen) and reversed transcribed using M-MLV reverse transcriptase. The cDNA was used as a template for PCR amplification with various 5′ primers within *SPRY4-IT1* and 3′ primers downstream in the *SPRY4* locus. Successful amplification was achieved with two forward primers: 5′ IT1 (gtagagatgggggtttcatcctgttg), which anneals to the first base of *SPRY4-IT1,* and *SPRY4-IT1* qPCR (gctgagctggtggttgaaaggaatc), which is located 305 bp within the *SPRY4-IT1* sequence. Successful amplification at the 3′ end was achieved with the reverse primer *SPRY4* Exon 3 (gtccgctttgggccggtgg), which is located 229 bp into the third exon of *SPRY4*. Amplification was unsuccessful using primers 500 bp downstream of this location. PCR products were cloned and sequenced. Sequences were aligned to the genomic template and verified using Vector NTi Advance and AlignX.

### Northern Blot Analysis

Total RNA was isolated from each cell line with TRIzol and concentrated. Aliquots of RNA (20 μg per sample) were mixed with equal volumes of 2× NorthernMax-Gly Loading Dye (with ethidium bromide), heated at 50°C for 30 min, and resolved on a 1% glyoxal agarose gel run at 65 V for 2 h 20 min in 1× running buffer. The gel was transferred to a nylon membrane (BrightStar Plus, Ambion) using downward capillary transfer, crosslinked using a UVP UV crosslinker at 1200 (UVP HL-2000 HybriLinker), prehybridized for 30 min at 42°C in 6 ml ULTRAhyb (Ambion), and then probed overnight at 42°C. Blots were processed with the BrightStar BioDetect kit (Ambion) and exposed to film. The following biotin-labeled DNA probes (IDT) were used at 166 nM: *SPRY4-IT1,* ctccactgggcatattctaaaa; *SPRY4* Exon 1, gatgttgcaaccactgcctgg; *SPRY4* Exon 3, catggctggtcttcacctggtc; and GAPDH, gggccatgaggtccaccaccc.

### Invasion Assay

A375 cells were trypsinized and reverse transfected using RNAiMax (Life Technologies) with 50 μM of the following siRNAs (Life Technologies): *SPRY4-IT1* Stealth RNAi 594, *SPRY4* Exon 1 Custom Select siRNA, lipin 2 Silencer Select siRNAs s18590 and s18591, and Universal Negative control (Cat #46-2001).

BD BioCoat™ Matrigel™ Invasion Chambers (24-well plates) were prepared by rehydrating the BD Matrigel™ matrix coating with 0.5 ml of serum-free Complete Tu medium for 2 h at 37°C in a 5% CO_2_ atmosphere. After rehydration, the solution was removed and 0.5 ml of Complete Tu medium containing chemoattractant (2% FBS) was added to the lower wells, and 0.5 ml of serum-free Complete Tu medium containing 5 × 10^4^ A375 cells transfected with siRNAs was added to the insert wells. Invasion assay plates were incubated for 2 days at 37°C in a humidified 5% CO_2_ atmosphere. Non-invading cells were removed by scrubbing the upper surface of the insert, and invading cells on the lower surface of the insert were stained with crystal violet. Each membrane was mounted on a microscope slide for visualization and analysis. Slides were scanned using a Scanscope digital slide scanner, and the migrated cells were enumerated using Aperio software. All experiments were performed with biological triplicates.

### Apoptosis Assay

A375 cells were trypsinized and reverse transfected with siRNAs as described above. Caspase activity was measured using the Caspase-Glo® 3/7 Assay kit (Promega) according to the manufacturer's recommendations, and samples were read on a GloMax luminometer (Promega).

### Polysome Fractionation

Cells grown in 10 cm dishes were gently removed with PBS and collected by centrifugation for 4 min at 100 × g. The cell pellet was resuspended in 500 μl of lysis buffer (100 mM KCl, 50 mM Tris-HCl pH 7.4, 1.5 mM MgCl_2_, 1 mM dithiothreitol (DTT), 1 mg/ml heparin, 1.5% NP-40, 100 μM cycloheximide, 1% aprotinin, 1 mM AEBSF, and 100U/ml of RNasin) and incubated on ice for 15 min. The lysate was centrifuged at 1000 × g and the infranatant lysate was removed from underneath the “fat cake”. The nuclei were pelleted by centrifugation in a microfuge for 10 min at 12,000 rpm. The resulting supernatant was carefully removed and applied to the top of a 15–60% sucrose gradient (in 100 mM KCl, 5 mM MgCl_2_, 20 mM HEPES pH 7.4, and 2 mM DTT). The gradients were centrifuged at 35,000 rpm for 3.5 h at 4°C in a Beckman SW41 rotor. Samples containing (i) free (unbound) mRNA, ribosomal subunits, and monosomes, and (ii) polysomes were recovered from the gradients using a Brandel density gradient fractionator equipped with an ISCO UA-6 flow cell set to 254 nm (a 70% sucrose solution containing orange G was slowly pumped into the bottom of the tube to displace the contents from top to bottom). Fractions were collected directly into an equal volume of TRIzol for isolation of RNA. The purity and integrity of RNA was determined with an Agilent Bioanalyzer prior to analysis by northern blotting.

### Affinity Purification of Endogenous SPRY4-IT1

A375 cells (10^8^ per sample) were grown to 80-90% confluency, the medium was discarded, and the plates were washed with PBS. The cells were trypsinized, pelleted at low speed, washed with PBS, and resuspended in 90 ml PBS (~10^6^ cells/ml). Cells were crosslinked by incubation in 1% formaldehyde at RT with rotation, and then quenched by the addition of glycine to a final concentration of 375 mM for 5 min. The cells were centrifuged at low speed, washed in PBS, and recentrifuged. The pellet was resuspended in Buffer A (1% NP-40, 1 mM DTT), homogenized, and spun at low speed. The pellet was resuspended in nuclei lysis buffer (1 mM DTT, 1× protease inhibitor, and 2 μl RNaseOut) at a volume of 1ml per 25 × 10^6^ cell equivalents. The lysate was incubated on ice, sonicated to yield 100-500 bp fragments (Duty Cycle, 20%; intensity, 10; cycles/burst, 200; and time, 8 min), and then centrifuged at high speed. The supernatant was removed and mixed with 0.5 M LiCl_2_, then 500 pmol/25 × 10^6^ cell equivalents of a *SPRY4-IT1* probe was added, and the sample was incubated at 37°C for 24 h. The probe consisted of a 25-bp sequence complementary to *SPRY4-IT1* constructed with a locked nucleic acid (LNA) backbone and a 5′-biotin label. Control samples contained probes complementary to the test probe sequence. A 2 ml aliquot of streptavidin-coated Dynabeads (Life Technologies) was washed twice with water and once with hybridization buffer, and then blocked with 800 μg yeast tRNA and 800 μg BSA for 1 h at 37°C. The beads were pelleted, washed twice in nuclear lysis buffer, and resuspended in 2 ml of the same buffer. Prepared beads were added to the nuclear lysate (125 μl per 500 pmol of probe), rotated at 37°C, and then washed twice in 1 ml nuclear lysis buffer and twice in 1 ml wash buffer (5 mM Tris-HCl pH 7.5, 0.5 mM EDTA, 1M NaCl). Beads from replicate samples were combined, washed twice in PBS, resuspended in PBS, incubated at 75°C for 5 min, and applied to a magnet. The isolated beads were incubated at 65°C overnight to reverse crosslinking, treated with 1 μg/ml RNase A, vortexed, and incubated at RT. The samples were then treated with proteinase K, resuspended in Buffer AL, and incubated at 56°C. Finally, RNA in the complexes was purified using the DNeasy Kit, eluted in RNase-free H_2_O, and qRT-PCR was performed to quantify *SPRY4-IT1*. The RNA-protein complexes were then subjected to LTQ Orbitrap Velos MS for analysis of *SPRY4-IT1*–associated proteins. Protein digestion, TiO_2_-based phosphopeptide enrichment, and ESI-MS/MS was performed at the proteomics core facility at Sanford-Burnham. Table S1 lists the identified *SPRY4-IT1*–binding proteins. RNA co-immunoprecipitation (RIP) was performed as previously described [36]. Lipin-2 antibody H-160 was from Santa Cruz (sc-134433).

### Real-time qRT-PCR

Quantitative PCR was carried out using TaqMan mRNA or SYBR Green mRNA assays with a 7500 Real-Time PCR System (Applied Biosystems/Life Technologies), in accordance with the manufacturer's protocols. SDS1.2.3 software (Applied Biosystems) was used for comparative Ct analysis, with 18S rRNA serving as the endogenous control. Primers for SYBR qPCR were as follows: *SPRY4-IT1* qPCR For, gctgagctggtggttgaaaggaatc; *SPRY4-IT1* qPCR Rev, gcttggcccacgatgacttgg; *SPRY4* Exon 1 qPCR For, ggcagtggttgcaacatcgccg; *SPRY4* Exon 1 qPCR Rev, tagccgccgctgtactcgcagac; *LPIN2* qPCR For, cgtctaagcagcccttctatgctgc; *LPIN2* qPCR Rev, acatgctccacgagctcactcagc; *LPIN1* qPCR For, ggagagctggtacaggaacatgcaaag; *LPIN1* qPCR Rev, gcagtggctctctccaaaaggtgaag; *GPAT3* qPCR For, caaggaggcctgactgaacttccc; *GPAT3* qPCR Rev, ccgtcctcttagctgagagatccattg; *DGAT1* qPCR For, caacaaggacggagacgccgg; *DGAT1* qPCR Rev, gatgccacggtagttgctgaagcc; *DGAT2* qPCR For, ctgcactgattgctggctcatcg; and *DGAT2* qPCR Rev, gaaagtagcgccacacagcccag.

### RNA-FISH Analysis

RNA-FISH was performed using an LNA-modified probe for human *SPRY4-IT1* (5′-Texas Red-TCCACTGGGCATATTCTAAAA) and a RiboMap *in situ* hybridization kit (Ventana Medical Systems, Inc.) on a Ventana machine. SPRY4-IT1-EE and vector-only cells were resuspended at 10^5^ cells/ml, and 100 μl was added to separate cloning rings on an autoclaved glass slide. The following day, the rings were removed, and the slide was washed in PBS and fixed in 4% paraformaldehyde and 5% acetic acid. After acid treatment using hydrochloride-based RiboClear reagent (Ventana Medical Systems) for 10 min at 37°C, the slide was treated with the ready-to-use protease 3 reagent, and subjected to a denaturing pre-hybridization step for 4 min at 80°C. The cells were then hybridized with 40 nM LNA-modified probe in RiboHybe hybridization buffer (Ventana Medical Systems) for 2 h at 58°C. The slide was washed 3 times for 4 min at 60°C; once at low stringency with 0.1× SSC (Ventana Medical Systems) and twice with 1× SSC. The slides were fixed in RiboFix and counterstained with DAPI in an antifade reagent (Ventana Medical Systems). Images were acquired using a Nikon A1R VAAS laser confocal microscope.

### 5′ RACE for SPRY4-IT1

5′ RACE was performed using the FirstChoice® RLM-RACE (RNA Ligase-Mediated RACE) kit (Ambion, Life Technologies). Total RNA was isolated from A375 cells with TRIzol, and 1 μg per sample was ligated to the 5′ RACE adapter using T4 RNA Ligase. This sample was then reverse transcribed using M-MLV reverse transcriptase. The samples were not treated with either calf intestinal alkaline phosphatase or tobacco acid pyrophosphatase and mRNA molecules thus retained both the 5′ 7-methyl cap and the 5′ phosphate. Multiple reverse primers were designed within the *SPRY4-IT1* sequence to complement the forward 5′ RLM-RACE primers. PCR reactions were performed using Phusion polymerase (Finnzymes), run out and purified from agarose gels, and cloned into pCR4-TOPO (Life Technologies) for sequence evaluation (Retrogen). The *SPRY4-IT1* RACE product was successfully acquired using the 5′ RACE inner primer (supplied with the FirstChoice® kit) and 5′ RACE *SPRY4-IT1* reverse primer, residing 385 bp into *SPRY4-IT1* (ctctctggggacgatgcagcatccgatgg).

## SUPPLEMENTARY MATERIAL, TABLES AND FIGURES








