# Supplementary material for: The Functional Characterization of Long Noncoding RNA SPRY4-IT1 in Human Melanoma Cells
Source: Oncotarget. 2014 Mar 26;5(19):8959–69. doi: 10.18632/oncotarget.1863 (PMC4253410; doi:10.18632/oncotarget.1863)
Supplement: Supplementary file 1 [file oncotarget-05-8959-s001.pdf]

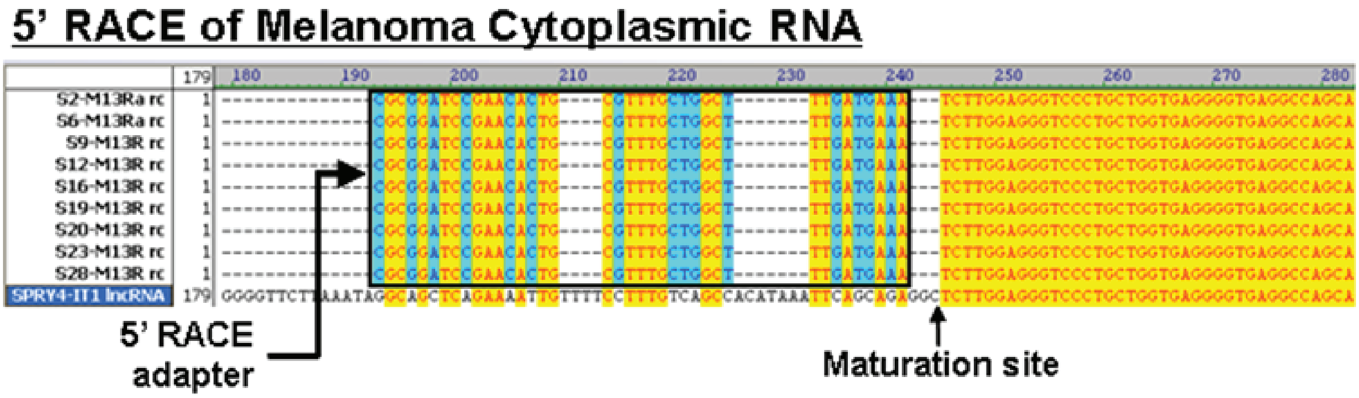

Figure S1. 5′ RACE Analysis of SPRY4-IT1 in the Cytoplasm
The 5′ RACE adapter and SPRY4-IT1 maturation site are indicated.

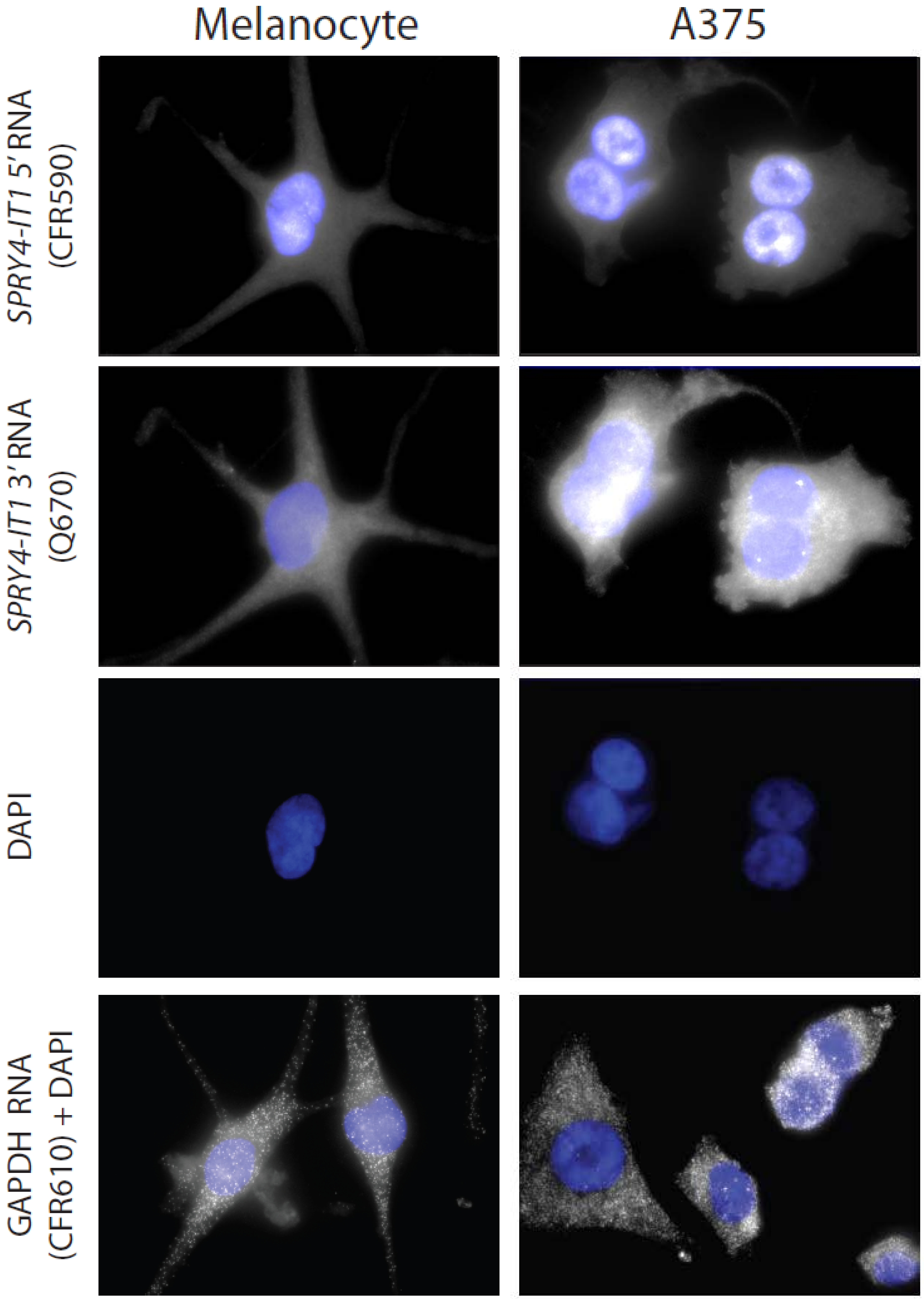

Figure S2. RNA-FISH Labeling Demonstrates the Existence of Nuclear and Cytoplasmic Forms of the SPRY4-IT1 Transcript

5′ and 3′ probes reveal nuclear and cytoplasmic expression, respectively, in melanocyte and A375 melanoma cells. GAPDH was probed as a control.

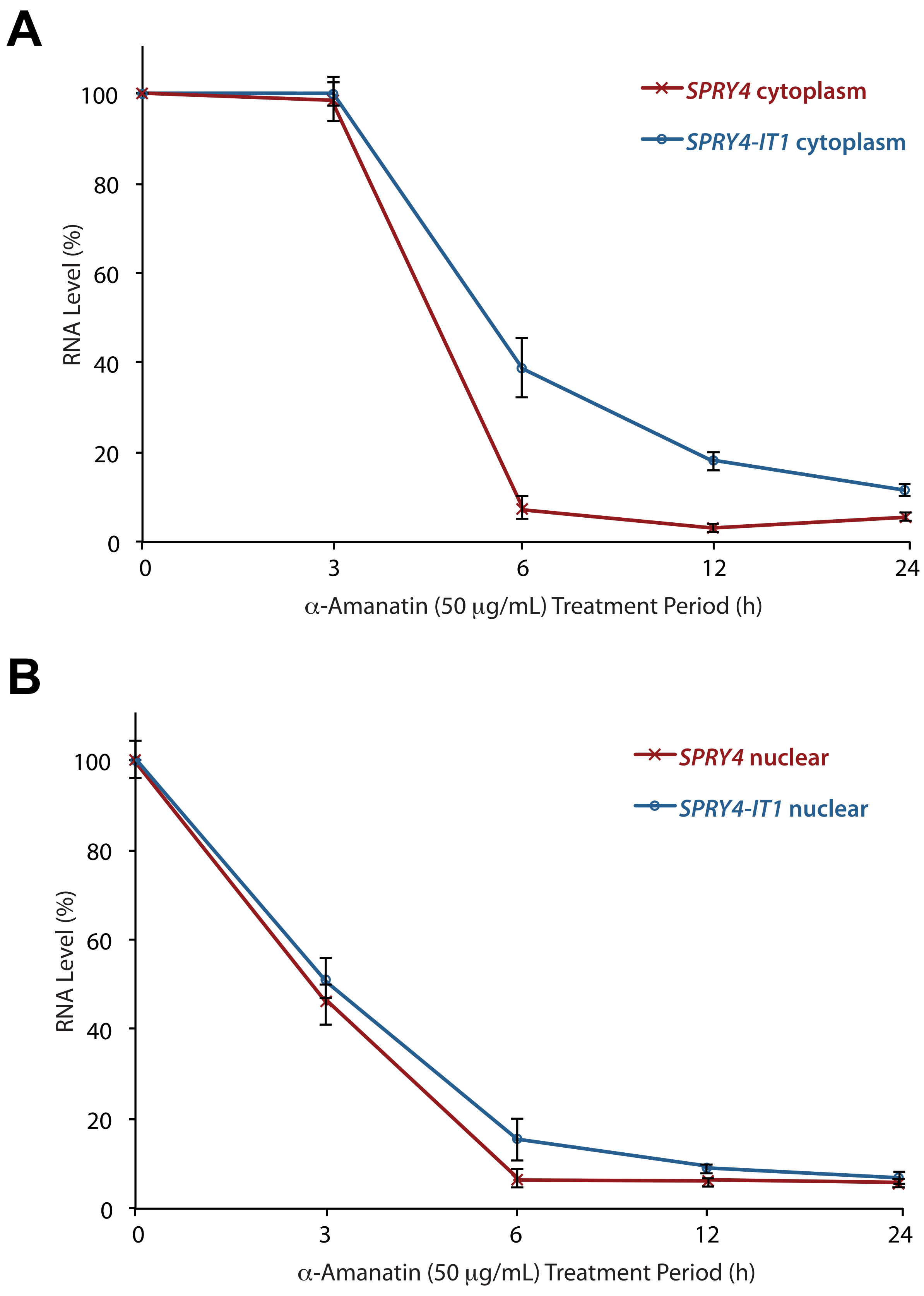

Figure S3. Relative Stability of SPRY4 and SPRY4-IT1 in Melanocytes

qRT-PCR analysis of SPRY4-IT1 and SPRY4 levels in the (A) cytoplasm and (B) nucleus of A375 cells treated with the RNA polymerase II inhibitor α-amanitin for up to 24 h. SPRY4-IT1 decays more rapidly than its host gene SPRY4; at 6 h, ~80% of SPRY4-IT1 transcripts have decayed compared with ~40% of SPRY4 transcripts.

A

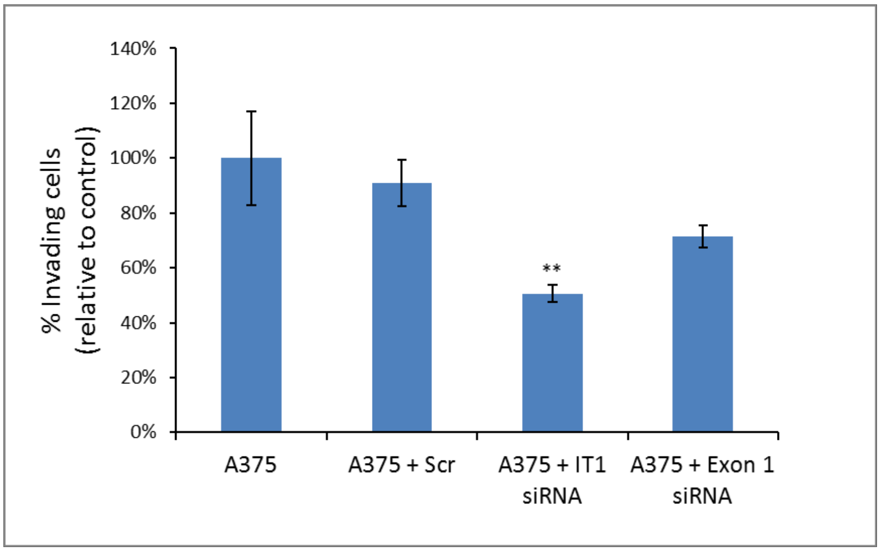

B

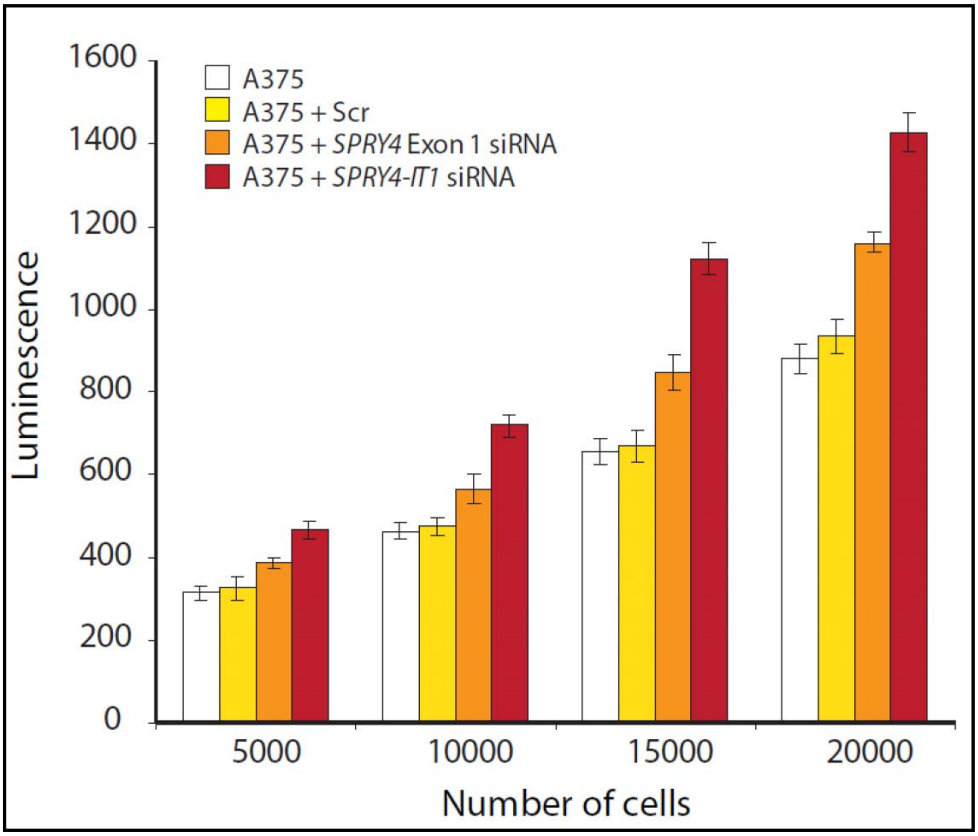

Figure S4. Effects of siRNA Targeting *SPRY4-IT1* and *SPRY4* Exon 1 on Cell Invasion and Apoptosis in A375 Cells

(**A**) Invasion efficiency and (**B**) apoptosis of A375 cells subjected to *SPRY4-IT1* and *SPRY4* exon 1 knockdown. Apoptosis was measured as an increase in caspase 3 activity in a luminescence-based assay.

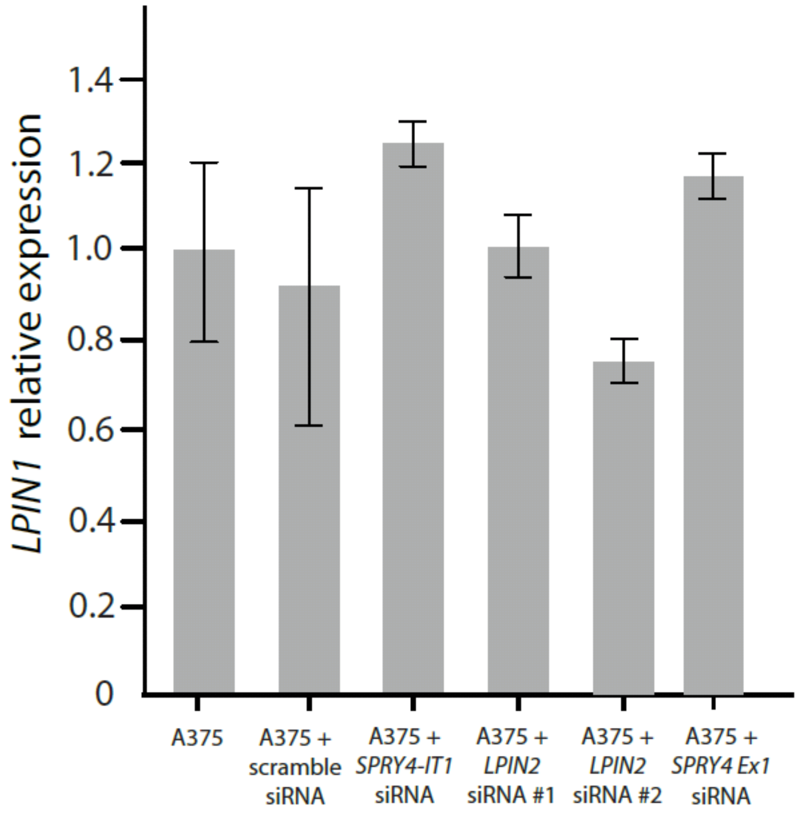

Figure S5. Lipin1 Relative Expression in SPRY4-IT1, SPRY4 Exon1, and Lipin2 Knockdown Cells
Knock-down of SPRY4-IT1, SPRY4 Exon1, and Lipin2, does not show lipin1 expression changes.

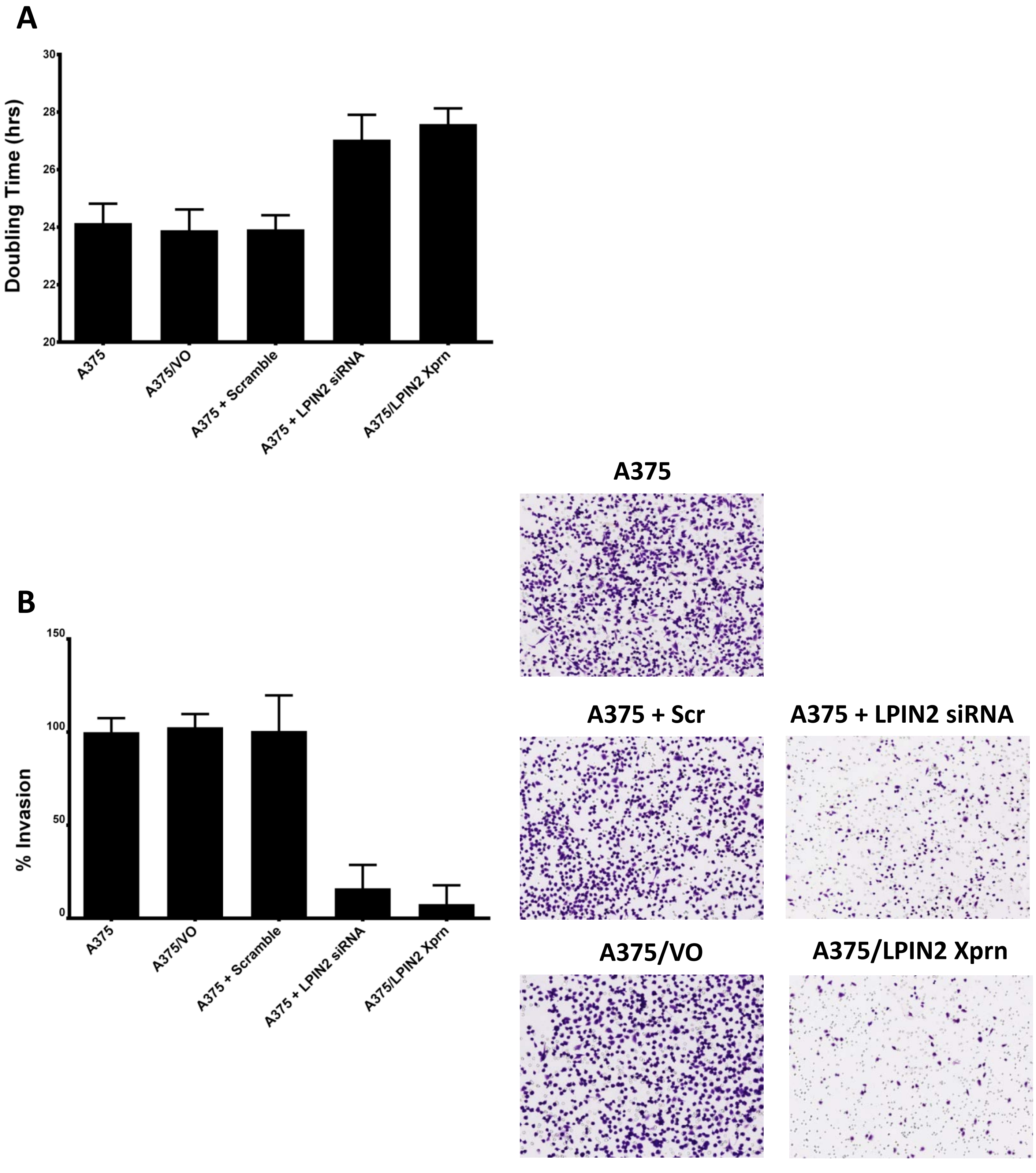

Figure S6. The effect of knock-down and over-expression of lipin 2 on cell growth and invasion in A375 cells

(A) The effect of SPRY4-IT1 knock-down or force-expression on cell growth

(B) The effect of SPRY4-IT1 knock-down or force-expression on cell invasion
