# Supplementary material for: The Functional Characterization of Long Noncoding RNA SPRY4-IT1 in Human Melanoma Cells
Source: Oncotarget. 2014 Mar 26;5(19):8959–69. doi: 10.18632/oncotarget.1863 (PMC4253410; doi:10.18632/oncotarget.1863)
Supplement: Supplementary file 2 [file oncotarget-05-8959-s002.pdf]

Table S1. SPRY4-IT1–binding Proteins Identified by Mass Spectrometry

Lipin 2 shows the highest spectral counts (83).

Number	Gene Symbol	Gene description	Subcellular location	Molecular function	Biological process	Peptide sequence	Nondegenerative sequence?	Instances observed	Total instances per gene
1	*ILF2*	Interleukin enhancer-binding factor 2	cytoplasm, nucleolus, nucleolus, nuc	ATP binding, DNA binding, double-st	immune response, positive regulatio	ILPTLEAVAALGNK	Yes	1	1
2	*GPR37*	Probable g-protein coupled receptor 37	endoplasmic reticulum, endoplasmic	G-protein coupled receptor activity,	G-protein coupled receptor signaling	LAVIWVGALLLALP	Yes	12	12
3	*SLC25A5*	ADP/ATP translocase 2	MMXD complex, integral to plasma r	adenine transmembrane transporter	adenine transport, chromosome seg	YFPTQALNFAFK	Yes	1	1
4	*RPLP1*	60S acidic ribosomal protein P1	cytoplasm, cytosol, cytosolic large rik	RNA binding, protein binding, structu	RNA metabolic process, cellular prot	AAGVNVEPFWPGLFAK	Yes	2	2
5	*IGF2BP1*	Insulin-like growth factor 2 mrna-binding protein 1	CRD-mediated mRNA stability compl	mRNA-UTR binding, mRNA;-UTR binc	CRD-mediated mRNA stabilization, R	MVIITGPPEAQFK	Yes	2	2
6	*RPS3*	40S ribosomal protein S3	cytoplasm, cytosol, cytosol, cytosolic	DNA-(apurinic or apyrimidinic site) ly	RNA metabolic process, activation of	FGFPEGSVELYAEK	No	2	4
						GGKPEPPAMPQPVPTA	Yes	2	
7	*RPS6*	40s ribosomal protein S6	cytoplasm, cytosol, cytosolic small ril	protein binding, protein kinase bindi	RNA metabolic process, T cell differe	DIPGLTDTTVPR	Yes	2	2
8	*LPIN2*	Phosphatidate phosphatase lipin2	ic reticulum, endoplasmic reticulum r	natidate phosphatase activity, transcr	sitive regulation of transcription from	TIFTPS[167]SVKK	Yes	19	102
						TIFTPSS[167]VKK	Yes	83	
9	*ALB*	Uncharacterized protein	basement membrane, cytoplasm, ex	DNA binding, antioxidant activity, ce	bile acid and bile salt transport, bile	KVPQVSTPTLVEVSR	Yes	3	3
10	*NACA*	Nascent polypeptide-associated complex subunit alpha	cytoplasm, nascent polypeptide-assc	DNA binding	interspecies interaction between org	IEDLSQQAQLAAAEK	Yes	2	2
11	*HNRNPCL1*	Heterogeneous nuclear ribonucleoprotein c-like 1	nucleus, ribonucleoprotein complex	RNA binding, nucleotide binding		VFIGNLNTLVVK	Yes	3	3
12	*TRAP1*	Heat shock protein 75 kda, mitochondrial	cellular_component, mitochondrion	ATP binding, nucleotide binding, tum	cellular response to oxidative stress,	GVVDSEDIPLNLSR	Yes	13	13
13	*TUBB2B*	Tubulin beta-2b chain	cytoplasm, cytoskeleton, microtubule	nucleotide binding, peptide binding	bule-based movement, mitosis, neur	AFLHWYT[181]GEGM[147]DEME	No	1	70
						AILVDLEPGTM[147]DSVR	No	15	
						AILVDLEPGTM[147]DSVR	No	1	
						AILVDLEPGTMDSVR	No	11	
						GHYTEGAELVDSVLDVVR	No	1	
						GHYTEGAELVDSVLDVVRK	No	5	
						INVYYNEATGNKYVPR	Yes	4	
						ISEQFTAM[147]FR	No	4	
						ISEQFTAMFR	No	1	
						LAVNM[147]VPFPR	No	10	
						LAVNMVPFPR	No	2	
						LHFFM[147]PGFAPLTSR	No	5	
						LHFFM[147]PGFAPLTSR	No	2	
						LTTPTYGDLNHLVSATM[147]SGVTTCLR	No	2	
						LTTPTYGDLNHLVSATMSGVTTCLR	No	2	
						M[147]SATFIGNSTAIQELFK	No	1	
						YLTVAAIFR	No	3	
14	*RPS8*	40S ribosomal protein S8	cytoplasm, cytosol, cytosolic small ril	structural constituent of ribosome, s	RNA metabolic process, cellular prot	IIDVVYNASNNELVR	Yes	2	2
15	*HIST1H1B*	Histone H15	chromosome, nucleosome, nucleus	DNA binding	nucleosome assembly	ATGPPVSELITK	Yes	1	1
16	*HSPE1*	10 kda heat shock protein, mitochondrial	cytoplasm, mitochondrial matrix, mi	ATP binding, chaperone binding, cha	activation of cysteine-type endopept	VVLDDKDYFLFR	Yes	2	2
17	*DPYSL2*	Dihydropyrimidinase-related protein 2	ondrion, neuronal cell body, protein	activity, acting on carbon-nitrogen (	tive regulation of glutamate secretio	GLYDGPVCEVSVTPK	Yes	2	4
						ILDLGITGPEGHVLSRPEEVEAEAVNR	Yes	1	
						MDENQFVAVTSTNAAK	No	1	
18	*VCL*	Isoform 1 of vinculin	mere, cytoplasm, cytoskeleton, cytos	tenin binding, beta-dystroglycan bind	esion, lamellipodium assembly, mor	AQQVSQGLDVLTAK	Yes	1	14
						AVGT[181]ANKS[167]T[181]VEGIQASVK	Yes	1	
						LVQAAQM[147]LQ	No	12	
19	*RPL7A*	60s ribosomal protein l7a	omal subunit, membrane fraction, po	protein binding, structural constituer	process, ribosome biogenesis, transl	AGVNTVTTLVENK	No	1	2
						LKVPPAINQFTQALDR	Yes	1	
20	*RPL24*	60S ribosomal protein L24	cytosol, cytosolic large ribosomal sul	RNA binding, structural constituent c	RNA metabolic process, cellular prot	AITGASLADIM[147]AK	Yes	2	12
21	*IGH@*	Putative matrix cell adhesion molecule-3				LTM[147]TRDTSTST	Yes	9	10
						VGPGAHS[167]QVQLVQS[167]GAEVK	No	1	
22	*NYNRIN*	Isoform 1 of protein nynrin	component, integral to membrane,	molecular_function, nucleic acid bindir	DNA integration, biological_process	ALELALLQGLLG	Yes	1	8
						LEVT[181]VSHVALTA	Yes	1	
						LEVTVS[167]HVALTA	Yes	5	
						LPFTFAGNLFMVPDDPLGR	Yes	1	
23	*RPSA*	Laminin receptor-like protein lamrl5	90S preribosome, cytoplasm, cytopla	protein binding, receptor activity, rib	RNA metabolic process, cell adhesio	AIVAIENPADVSVISSR	Yes	1	1
25	*DDX17*	Probable atp-dependent rna helicase ddx17 isoform 3	nucleus	ATP binding, ATP-dependent helicas	RNA processing	ELAQQVQQVADDYGK	Yes	1	1
