# Supplementary material for: The Functional Characterization of Long Noncoding RNA SPRY4-IT1 in Human Melanoma Cells
Source: Oncotarget. 2014 Mar 26;5(19):8959–69. doi: 10.18632/oncotarget.1863 (PMC4253410; doi:10.18632/oncotarget.1863)
Supplement: Supplementary file 3 [file oncotarget-05-8959-s003.pdf]

Table S2. Lipidomic Analysis of A375 Cells After Knockdown of SPRY4-IT1
SPRY4-IT1 knockdown decreases cellular diacylglycerol (DAG) levels by ~10% and increases triacylglycerol levels by ~15%, consistent with the observed effects of SPRY4-IT1 knockdown on acyl-coA:glycerol-3-phosphate acyltransferase 3 (GPAT3) and diacylglycerol O-acyltransferase 2 (DGAT2) expression (Fig. S6).

Lipidomic analysis of A375 cells subjected to SPRY4-IT1 knock-down

Lipid Species	A375	IT1	Percentage (%) (IT1/A375)
**Acyl Carnitine (CAR) (pmol/mg protein)**	0.255286	0.460055	80.21%
**Cardiolipin (CL) (nmol/mg protein)**	4.826375	2.595352	-46.23%
**Ceramide (CER) (nmol/mg protein)**	0.929514	1.056996	13.71%
**Diacylglycerol (DAG) (nmol/mg protein)**	1.898592	1.704089	-10.24%
**Fatty Acyl Chains in TAG (FA) (nmol/mg protein)**	51.30639	58.61157	14.24%
**Lyso Phosphatidylcholine (LPC) (nmol/mg protein)**	1.224603	1.01981	-16.72%
**Phosphatidic acid (PA) (nmol/mg protein)**	1.026541	0.639301	-37.72%
**Phosphatidylcholine (PC) (nmol/mg protein)**	49.64564	31.98054	-35.58%
**Phosphatidylethanolamine (PE) (nmol/mg protein)**	30.19263	34.19713	13.26%
**Phosphatidylglycerol (PG) (nmol/mg protein)**	2.886709	3.42792	18.75%
**Phosphatidylinositol (PI) (nmol/mg protein)**	12.47999	9.012531	-27.78%
**Phosphatidylserine (PS) (nmol/mg protein)**	15.45311	11.37352	-26.40%
**Sphingomyelin (SM) (nmol/mg protein)**	12.08027	10.85326	-10.16%
**Triacylglycerol (TAG) (nmol/mg protein)**	17.69247	20.35036	15.02%
