# Supplementary material for: The Functional Characterization of Long Noncoding RNA SPRY4-IT1 in Human Melanoma Cells
Source: Oncotarget. 2014 Mar 26;5(19):8959–69. doi: 10.18632/oncotarget.1863 (PMC4253410; doi:10.18632/oncotarget.1863)
Supplement: Supplementary file 4 [file oncotarget-05-8959-s004.pdf]

Table S3.
Lipidomic analysis Of HEM-l vs A375 Melanoma cells

Lipid Species	HEM-l	A375	Percentage (%) (A375/HEM-l)
**Acyl Carnitine (CAR) (pmol/mg protein)**	19.48	37.43	92.13%
**Cardiolipin (CL) (nmol/mg protein)**	0.26	2.11	699.67%
**Ceramide (CER) (nmol/mg protein)**	1.26	0.74	-41.17%
**Diacylglycerol (DAG) (nmol/mg protein)**	6.40	5.46	-14.70%
**Fatty Acyl Chains in TAG (FA) (nmol/mg protein)**	42.79	125.15	192.45%
**Lyso Phosphatidylcholine (LPC) (nmol/mg protein)**	0.62	1.01	63.08%
**Phosphatidic acid (PA) (nmol/mg protein)**	1.26	1.04	-17.77%
**Phosphatidylcholine (PC) (nmol/mg protein)**	27.23	40.85	50.01%
**Phosphatidylethanolamine (PE) (nmol/mg protein)**	50.61	32.79	-35.21%
**Phosphatidylglycerol (PG) (nmol/mg protein)**	1.74	1.79	2.98%
**Phosphatidylinositol (PI) (nmol/mg protein)**	9.97	11.28	13.09%
**Phosphatidylserine (PS) (nmol/mg protein)**	19.55	10.15	-48.05%
**Sphingomyelin (SM) (nmol/mg protein)**	12.51	3.67	-70.64%
**Triacylglycerol (TAG) (nmol/mg protein)**	11.27	32.75	190.56%
